# Recruiting and distributing eye health workers

**Published:** 2018-07-31

**Authors:** Suzanne Gilbert, Daksha Patel

**Affiliations:** 1 Senior Director: Innovation & Sight Programs, Seva Foundation, Berkeley, California USA.; 2 E-learning Director: International Centre for Eye Health, London School of Hygiene and Tropical Medicine, London, UK.

**Recruitment of the right people to the right places requires local investment.**

**Figure:**
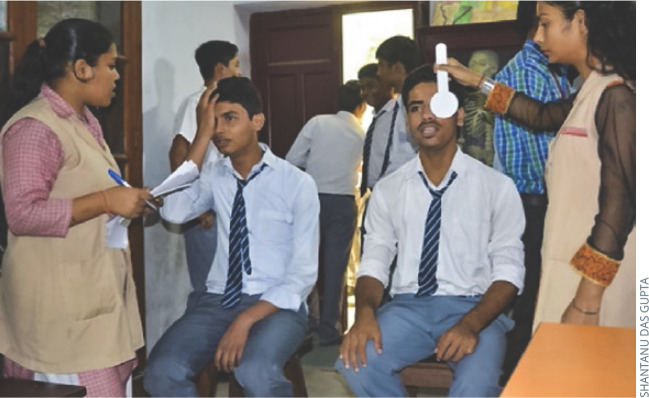
Testing visual acuity at a school screening camp. INDIA

As with any market, the eye health labour market facilitates an exchange between the **demand** for trained personnel to meet the health needs of the population and the **supply** (or availability) of trained personnel.

The labour market in each country is influenced by a number of factors such as:

- Who is recruiting (private or public sector)?
- Where is the work required? (Urban or rural, or by level of service? For example, community level, district level or tertiary level)
- How many jobs are available, and what skills do they require? Are these new jobs, or replacement for retired personnel?
- How many skilled personnel are available to fill these posts? Are they graduates or transfers?
- What are the terms and conditions for the employment?

Recruitment of the right people to right places in eye health are influenced and challenged by a number of factors:

- The number entering (from education or migration) into the labour market (the supply)
- Where the jobs are (which should ideally correspond with where the eye health needs are)
- The conditions of employment
- Policies on recruitment and distribution
- Numbers exiting the services (due to retirement, death or migration), which makes positions available.

## The number entering the labour market

The number of eye health professionals trained annually are insufficient to meet the need. Data from the Vision Loss Expert Group (VLEG)^1^ indicates the critical shortages experienced, and their impact on service provision. For example, fewer than 1% of the world's ophthalmologists are available to meet the needs of 4.8 million blind in Africa, and critical human resource shortages are experienced across all professional groups.

Even if there are enough ophthalmologists, it can be a challenge to recruit enough nurses and allied ophthalmic personnel to achieve the right balance of skills in an eye team. This often requires local solutions, such as the training programme for local women in India described by Shantanu Gupta (p. 45).

Governments must plan and invest in the recruitment of new eye personnel, or risk the possibility that promising candidates will choose to work in private health care or leave the country in search of better jobs.

## Distribution based on health needs

In the ideal setting, distribution of eye health workers would be based on where the demand is. In most low- and middle income countries, the eye health needs within rural settings are much higher than in urban settings, but attract very few eye health workers,^2^ often due to the inadequate working conditions.

Health needs are also changing, and the range of specialisations also need to be considered when distributing the skilled workforce. For example, the growing burden of diabetic retinopathy will require personnel for the establishment of services for screening, grading and treatment.

## Conditions of employment

Entry into employment needs to be balanced with how many are leaving the service; it is not always about creating new posts. Exit points may be due to death, retirement, migration or even a change from full-time to part-time roles. Employment conditions also need to be considered when trying to attract the right people to the right places (pp. 48–50).

## Policies on recruitment and distribution

Policies at the regional, national and local level need to evolve based on changes within the labour market, such as the migration of eye care workers to high-income countries.

Planning health workforce recruitment is a complex process. IAPB Africa is currently taking action to develop a harmonised, competency-based training curriculum to improve the distribution of skills in the eye team. The usefulness of mandatory placements to rural districts, and/or bridging the gaps through outreach services, need to identified by each country. Khumbo Kalua describes how this was done in Malawi (p. 47).

Recruitment must be carefully planned. Investment in hiring, placement and appropriate working conditions are essential to achieve universal access to good quality eye care.
